# Structure and stability in the community with the endangered plant *Heritiera parvifolia* Merr. (Malvaceae) in lowland rainforest in Diaoluo mountain of Hainan Island, China

**DOI:** 10.3389/fpls.2025.1578361

**Published:** 2025-06-13

**Authors:** Naiyan Shang, Shaocui He, Dongling Qi, Xiaobo Yang, Donghai Li, Rentong Liu, Chunyan Du, Xin Su, Tianyun Qi

**Affiliations:** ^1^ School of Ecology, Hainan University, Haikou, China; ^2^ Rubber Research Institute, China Academy of Tropical Agricultural Sciences, Haikou, China

**Keywords:** plant community, species diversity, stand factor, environmental factor, human interference, National Park of Hainan Tropical Rainforest

## Abstract

**Introduction:**

Endangered plants are crucial for ecosystem stability, influencing forest community structure. However, commercial logging and the expansion of economic forests have led to tropical forest habitat degradation and fragmentation.

**Methods:**

To assess the community structure and stability of *Heritiera parvifolia*, an endangered plant, in the Diaoluo Mountain zone of National Park of Hainan Tropical Rainforest in China, a field study was conducted across 20 plots. The analysis utilized species diversity indices, principal component analysis, ward clustering, linear regression, and one-factor analysis of variance.

**Results:**

The results showed high species richness in the community, with 78 families, 196 genera, and 302 species. Dominant families include Rubiaceae, Euphorbiaceae, and Lauraceae. The species richness, Shannon index, and Simpson index for the arbor and shrub layers were significantly higher than those of the herbaceous layer (*P* < 0.001), while the Pielou index was also higher (*P* < 0.01). Cluster analysis indicated that plots with less human disturbance (Group I) had greater stability than those with more disturbance (Group II). The stability index positively correlated with stand density, proportion of mature trees, average diameter at breast height and average tree height of dominant trees (*P* < 0.05), all of which were key factors in community stability. Stability was also significantly influenced by altitude and slope aspect, with significant differences observed between altitudes of 200–299 m and 400–499 m (*P* < 0.05), and between southeast slopes and other aspects (*P* < 0.05).

**Discussion:**

This study highlights the factors affecting the stability of *H. parvifolia* communities, providing insights for conservation, biodiversity protection, and rainforest restoration.

## 1 Introduction

Tropical rainforests are the most biodiverse ecosystems on Earth, supporting a wide variety of plant and animal species. They are crucial for ecosystem stability and water conservation (Razafindratsima et al., 2018; Dang et al., 2019; Hansen et al., 2020). However, commercial logging and land-use changes have drastically reduced their area, accelerating the extinction of many rare and endangered species (Noël et al., 2013; Lian et al., 2024). The Hainan tropical rainforest, home to 173 nationally protected plant species across 83 genera and 53 families, represents China’s richest tropical flora (Li et al., 2024). The lowland rainforest, in particular, holds the highest diversity of endangered species, underscoring its ecological importance and conservation value. However, it has suffered extensive damage from commercial logging and the expansion of cash crop cultivation, such as *Areca catechu* and *Hevea brasiliensis*, leading to significant forest area loss. The lowland rainforest, in particular, with its favorable water and temperature conditions and easy accessibility, has suffered the most extensive damage (Du et al., 2024). The population of endangered species has sharply declined due to habitat degradation, reduced survival capacity, and external disturbances (Xu and Zang, 2023; Ali et al., 2024). Understanding the species composition, community structure, and stability of endangered plant communities in these rainforests is essential for their conservation, restoration, and effective management in national parks.

Current research on tropical forest community structure and stability focuses on functional diversity, ecological interaction networks, disturbance responses, and recovery strategies (Mafakheri et al., 2022; Monteiro et al., 2025; Zhang et al., 2023). Trees dominate the competition for community resources in tropical rainforests, influencing light distribution within the canopy (Chou et al., 2018). This creates distinct functional groups among understory plants, such as shade-tolerant palms. The proportion of dominant trees serves as a key indicator of community stability. Tropical rainforests are highly species-rich, and terrain factors—such as light, temperature, water availability, and soil conditions—contribute to habitat heterogeneity, fostering complex plant, animal, and microbial interactions (Dearborn and Danby, 2017; Chen et al., 2021). However, high species diversity does not always correlate with functional diversity, as some distantly related species may exhibit functional redundancy due to convergent evolution (Radford-Smith et al., 2024). In harsh conditions, species can compensate for functional losses, supporting overall community resilience. Conversely, human activities like illegal logging and agricultural expansion undermine rainforest stability (Yirga et al., 2019). Secondary forests regenerating after logging often face challenges due to intense interspecific competition, primarily from shrubs, hindering community succession (Rozendaal et al., 2019). Species diversity, terrain, forest regeneration, and human disturbance all influence community stability (Wales et al., 2020; Sadia et al., 2024). Endangered plants are crucial for ecosystem functioning and ecological restoration (Planchuelo et al., 2019), making the structural and stability characteristics of their communities a priority for research.

**Figure 1:**
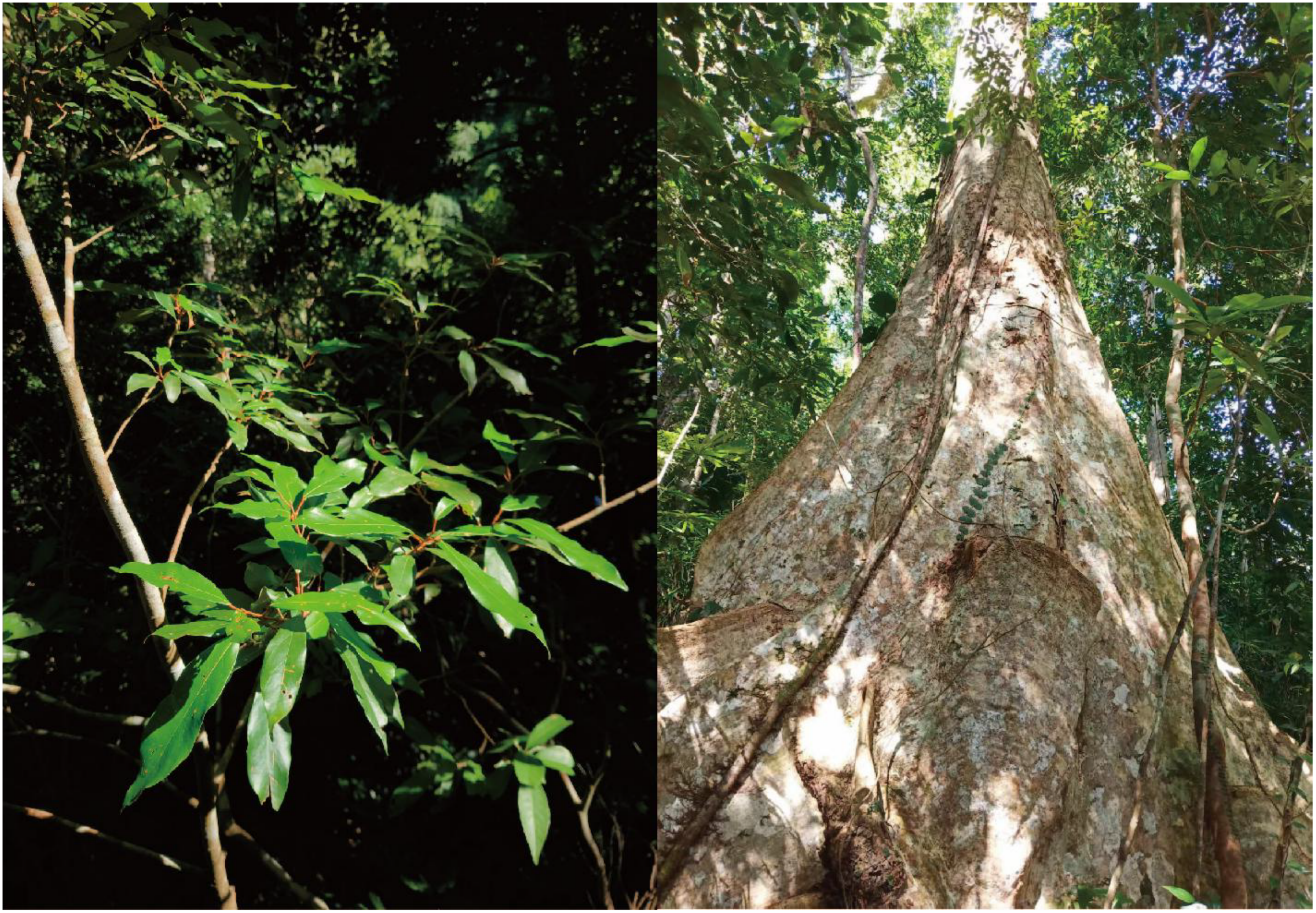
*Heritiera parvifolia* in the Diaoluo Mountain area of National Park of Hainan Tropical Rainforest, China.

*Heritiera parvifolia*, a national key second-level protected plant in China (Li et al., 2024), is a dominant species in the lowland rainforest of southeastern Hainan Island, playing a key role in forest development. The Diaoluo Mountain area of National Park of Hainan Tropical Rainforest contains the region’s most pristine rainforest, with healthy, widely distributed populations of *H. parvifolia* (**Figure 1**). Despite its ecological value and resistance to corrosion, this species has suffered from habitat degradation due to logging and land reclamation. Additionally, reproductive challenges, such as low seed production and rooting difficulties (Shang et al., 2025), have led to a decline in its population. Research on *H. parvifolia* has mainly focused on interspecific relationships, provenance breeding, and genome sequencing (Xin et al., 2018; Shang et al., 2025), but no studies have addressed both community structure and stability in the wild.

This study examines *H. parvifolia* as a focal species to analyze its community’s species composition and vertical structure. Using twenty indicators to characterize community structure and environmental conditions, the study employs principal component analysis (PCA) to assess community stability. The research aims to answer the following questions: (1) How do species composition and structural characteristics influence community stability? (2) Which factors are most closely related to community stability? (3) How do different altitudes, slopes, and slope aspects affect stability?

## 2 Materials and methods

### 2.1 Study area and field survey

**Figure 2:**
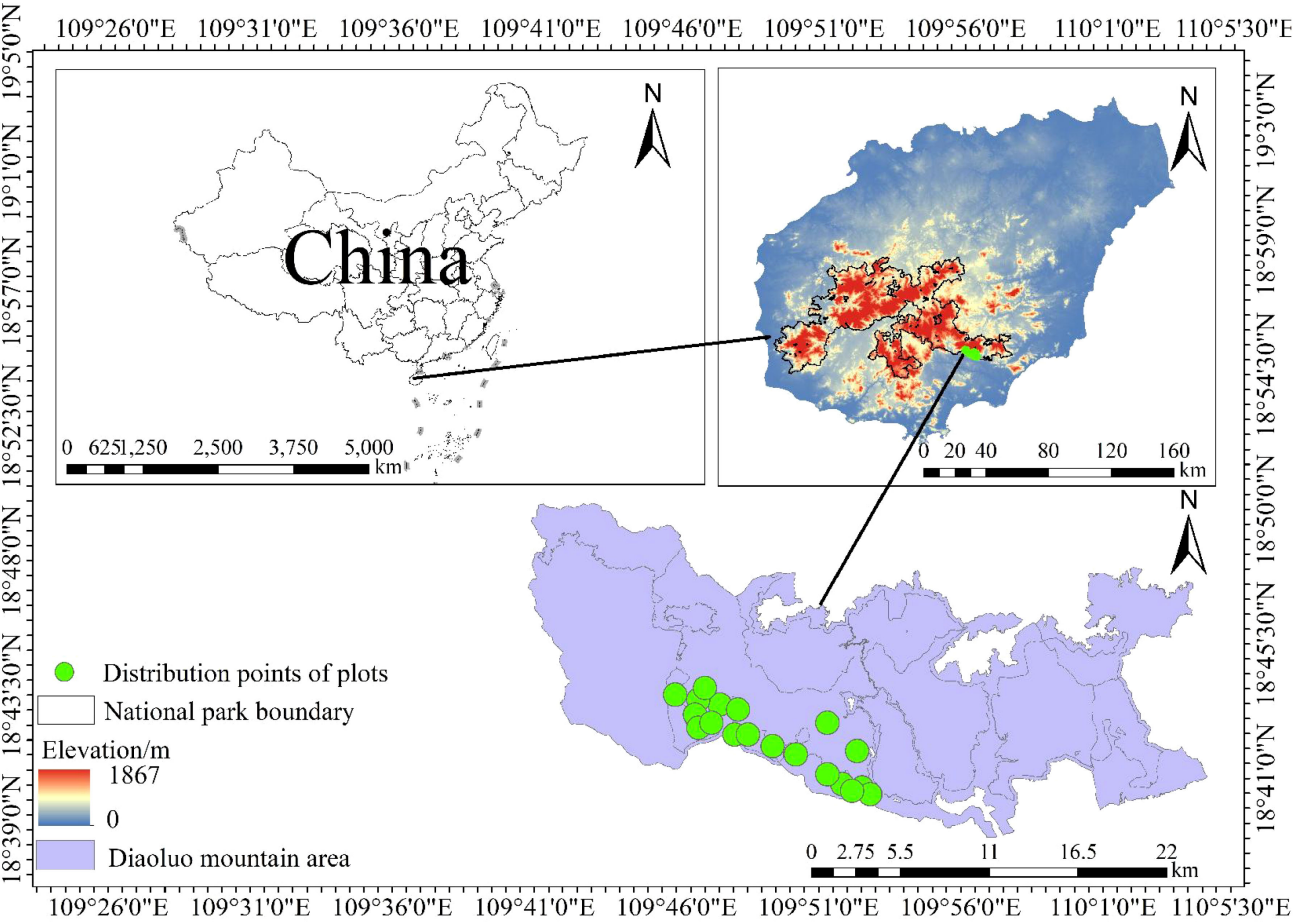
Sampling points for *Heritiera parvifolia* survey in Diaoluo Mountain of Hainan Island, China.

The study was conducted in the Diaoluo Mountain area of Hainan Tropical Rainforest National Park, located at the intersection of Lingshui, Baoting, and surrounding counties (18°43′—18°58′N, 109°45′—110°30′E, **Figure 2**). This region has an average annual temperature of 24.6°C and annual precipitation ranging from 1,870 to 2,760 mm, spanning three temperature zones and two rainfall regions in Hainan. The vegetation is diverse, including lowland rainforest, mountain rainforest, and alpine cloud forest (Shang et al., 2025).

Twenty 20m × 20m sample plots were set up within the lowland rainforest at altitudes of 200–700 m. Each plot contained five 1m × 1m herbaceous quadrats, covering a total area of 8,000 m². Plot locations were carefully selected to minimize spatial autocorrelation, with adjacent plots placed over 100 m apart. Basic plant data (e.g., DBH, tree height, crown width) were recorded. Trees with DBH < 2.5 cm were categorized as seedlings, 2.5 cm ≤ DBH < 7.5 cm as saplings, and DBH ≥ 7.5 cm as adults (Zhang et al., 2019). Altitude, slope, and slope aspect were also recorded. The community was stratified into arbor (H ≥ 5 m), shrub (0.5 m ≤ H < 5 m), and herbaceous (H < 0.5 m) layers. Diversity indices such as species richness, Shannon, Simpson, and Pielou were calculated (Asigbaase et al., 2019).

### 2.2 Stability evaluation index system

Twenty indicators representing community structure and environmental conditions were selected to form an evaluation system, categorized into four areas: species diversity, forest stand structure, terrain, and forest regeneration. The species diversity index included measures from the arbor, shrub, and herbaceous layers (species richness, Shannon, Simpson, Pielou). Forest stand structure comprised stand density, average DBH, and tree height of dominant species. Terrain factors included slope and slope aspect, while forest regeneration was assessed by the proportion of seedlings, saplings, and adult trees.

### 2.3 Principal component analysis of community stability

PCA was used to assess community stability by considering both vegetation and environmental factors. Eigenvalues and component contributions were analyzed to interpret stability, with higher values indicating greater explanatory power. Indicators were standardized, and principal components were selected based on a cumulative contribution rate exceeding 85%. The scores for each plot were calculated and summed to derive a stability index (Mikha et al., 2024).

### 2.4 Community stability clustering

Ward clustering was applied to the stability indices of the twenty plots, and the silhouette coefficient method was used to determine the optimal number of clusters. A silhouette value closer to 1 indicates better clustering quality (Zhang et al., 2015).

### 2.5 Relationship between community stability and key indicators

Univariate linear regression was employed to assess the relationship between significant indicators from the first principal component and the stability index. Model fit was evaluated using *R*², and 95% confidence intervals were provided.

### 2.6 Analysis of terrain’s impact on community stability

One-factor analysis of variance was used to assess the impact of different altitudes, slopes, and slope aspects on community stability, with statistical significance set at *P* < 0.05. Altitudes were categorized into five levels, namely I (200–299 m), II (300–399 m), III (400–499 m), IV (500–599 m) and V (600–699 m), respectively. Slopes into five degrees of incline, namely S1 (0°–9°), S2 (10°–19°), S3 (20°–29°), S4 (30°–39°) and S5 (40°–49°), and slope aspects into north, east, southeast, southwest, and northwest. respectively.

### 2.7 Data analysis and software

Data were organized in Excel 2016, PCA and regression analysis were conducted in SPSS 26.0, ward clustering and silhouette coefficient analysis were performed in R 4.3.2, and ANOVA and figures were generated in Origin 2021.

## 3 Results

### 3.1 Community structure and species composition in the community with *Heritiera parvifolia*

**Figure 3:**
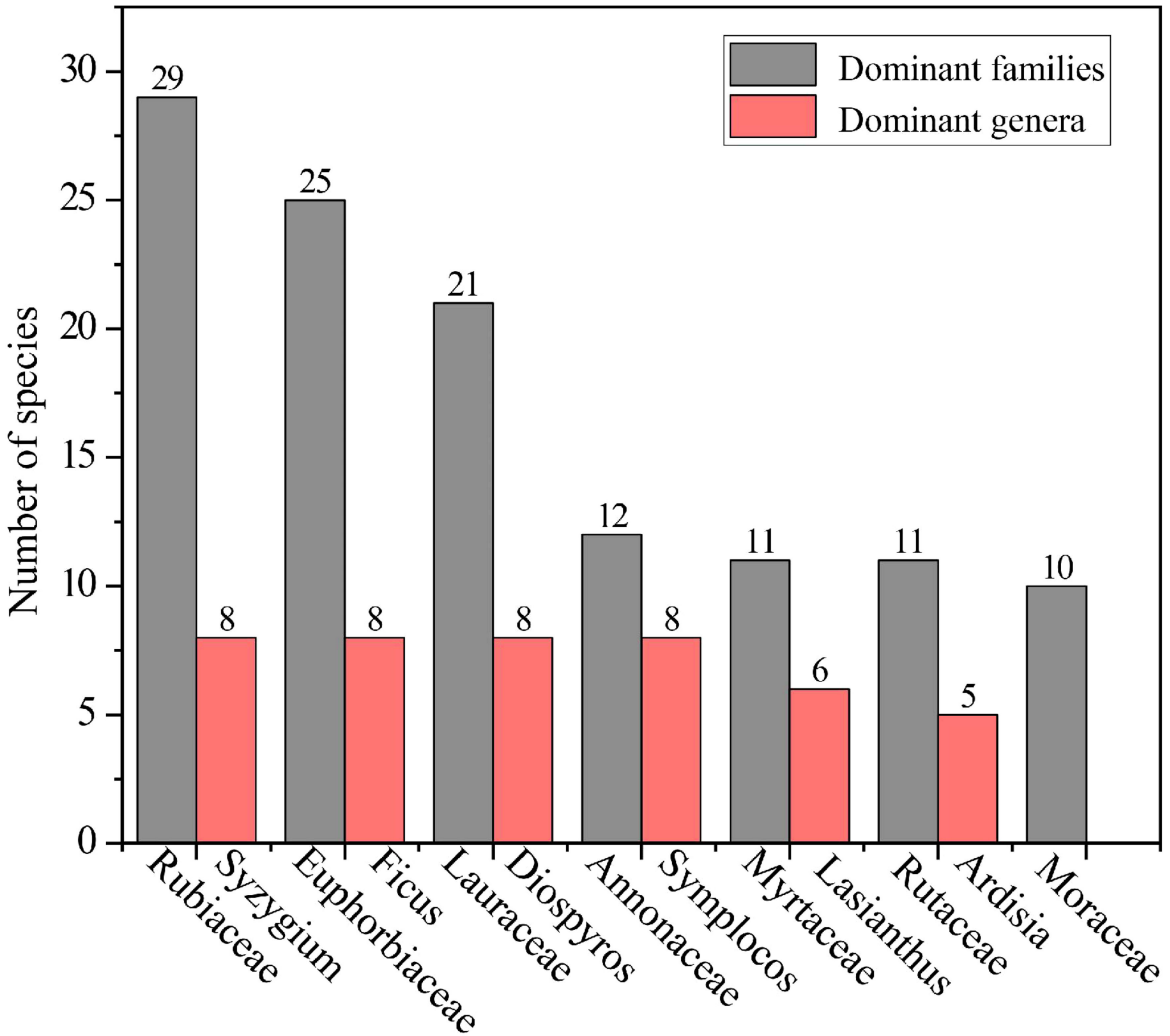
Dominant families (10 or more species) and genera (5 or more species) in the community.

The community in Hainan’s Diaoluo Mountain area is rich in plant species, with 302 species across 195 genera and 77 families (**Table 1**). It includes 4 families, 5 genera, and 6 species of ferns, and 73 families, 190 genera, and 296 species of seed plants. Among the seed plants, there is 1 gymnosperm family, genus, and species, and 72 families, 189 genera, and 295 angiosperm species. Dominant families include Rubiaceae (29 species), Euphorbiaceae (25 species), Lauraceae (21 species), Annonaceae (12 species), Myrtaceae (11 species), Rutaceae (11 species), and Moraceae (10 species). Prominent genera include *Syzygium* (8 species), *Ficus* (8 species), *Diospyros* (8 species), *Symplocos* (8 species), *Lasianthus* (6 species), and *Ardisia* (5 species) (**Figure 3**).

**Table 1:** Species composition in the community.

Plant taxa	No. of families	No. of genera	No. of species
Ferns	4	5	6
Gymnosperms	1	1	1
Angiosperms	72	189	295
Total	77	195	302

In the arbor layer, the main dominant species are *Vatica mangachapoi*, *H. parvifolia*, *Lithocarpus silvicolarum*, and *Alphonsea monogyna*. The shrub layer features species such as *Psychotria asiatica*, *Chunia bucklandioides*, and *Garcinia oblongifolia*, while the herbaceous layer includes *Pronephrium simplex* and *Schizostachyum hainanense* (**Table 2**).

**Table 2:** Importance value of dominant species in the community.

Levels	Species name	*IV*/%
Arbor	*Vatica mangachapoi*	4.76
	*Heritiera parvifolia*	4.27
	*Lithocarpus silvicolarum*	2.12
	*Alphonsea monogyna*	2.05
Shrub	*Heritiera parvifolia*	3.85
	*Vatica mangachapoi*	3.22
	*Psychotria asiatica*	2.92
	*Chunia bucklandioides*	2.65
	*Garcinia oblongifolia*	1.51
Herbaceous	*Pronephrium simplex*	7.67
	*Schizostachyum hainanense*	6.37

*IV*, importance value.

### 3.2 Species diversity characteristics of different vertical forest layers

**Figure 4:**
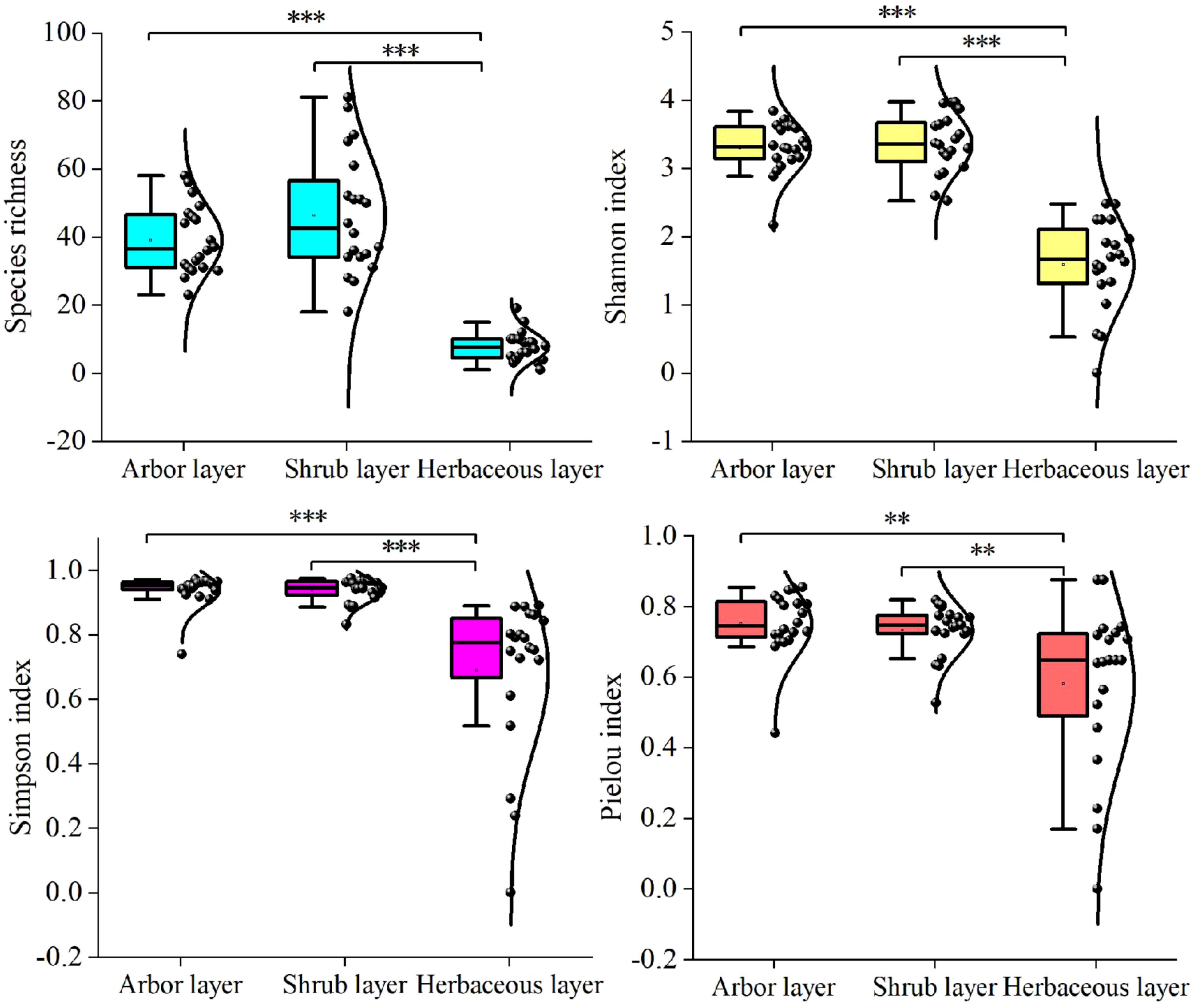
Species diversity in the community at different layers. The star represents a significant correlation (**P<0.01, ***P<0.001).

Species richness, Shannon index, and Simpson index for the arbor and shrub layers were significantly higher than in the herbaceous layer (*P* < 0.001), with the Pielou index also significantly higher in the arbor and shrub layers (*P* < 0.01) (**Figure 4**). This indicates that species richness and diversity are lowest in the herbaceous layer.

### 3.3 Principal component analysis of community stability

PCA of twenty survey indicators revealed that the first five principal components accounted for 88.91% of the total variance, surpassing the 85% threshold (**Table 3**). The first three principal components were particularly influential. The first component explained 27.34% of the variance, with key positive influences from stand density (0.94), proportion of arbor adults (0.94), average DBH (0.83), and average height of dominant trees (0.92). Negative influences included proportion of arbor seedlings (-0.86), species richness (-0.77), and Shannon index (-0.60) in the shrub layer. The second component (25.44%) was strongly influenced by species diversity indices in both the shrub and arbor layers, particularly the Shannon index (0.76), Simpson index (0.93), and Pielou index (0.88) in the shrub layer, and similar indices in the arbor layer. The third component (17.63%) was primarily influenced by diversity indices in the herbaceous layer, with the highest impacts from Shannon index (0.95), Simpson index (0.92), and Pielou index (0.87). The fourth component was most influenced by the proportion of arbor saplings (-0.93) and slope (0.79), while the fifth component was affected by slope aspect (-0.76) and species richness in the arbor layer (0.68).

**Table 3:** Principal component eigenvalue, contribution rate and indicator loading matrix.

Index	Component matrix				
	1	2	3	4	5
Arbor layer richness	-0.19	0.44	0.31	-0.15	0.68
Shrub layer richness	-0.77	0.44	0.03	0.30	0.21
Herbaceous layer richness	-0.33	0.04	0.75	0.31	0.18
Arbor layer shannon index	0.07	0.84	0.27	0.03	0.38
Shrub layer shannon index	-0.60	0.76	0.10	0.04	0.11
Herbaceous layer shannon index	-0.19	0.18	0.95	0.06	0.08
Arbor layer simpson index	0.20	0.89	0.24	0.07	0.02
Shrub layer simpson index	-0.20	0.93	0.10	-0.14	-0.02
Herbaceous layer simpson index	-0.16	0.24	0.92	-0.05	-0.01
Arbor layer pielou index	0.27	0.83	0.08	0.34	0.03
Shrub layer pielou index	0.05	0.88	0.20	-0.26	-0.04
Herbaceous layer pielou index	-0.05	0.26	0.87	-0.12	-0.06
Stand density	0.94	0.02	-0.18	0.13	-0.22
The average DBH of dominant trees	0.83	0.28	-0.20	0.25	-0.12
The average height of dominant trees	0.92	0.15	-0.14	0.00	0.06
Slope	0.22	-0.18	0.16	0.79	0.30
Slope aspect	0.23	0.00	0.08	-0.15	-0.76
The proportion of arbor seedlings	-0.86	0.06	0.10	0.43	0.04
The proportion of arbor saplings	0.11	-0.08	0.07	-0.93	0.11
The proportion of arbor adults	0.94	-0.01	-0.16	0.16	-0.13
Eigenvalues	5.47	5.09	3.53	2.24	1.46
Proportion/%	27.34	25.44	17.63	11.21	7.29
Cumulative/%	27.34	52.78	70.41	81.62	88.91
Weight coefficient	0.31	0.29	0.20	0.13	0.08

The underlined numbers in the table are positive or negative indicators that have a large impact on the principal components.

### 3.4 Evaluation of community stability in different plots

The community stability scores of the twenty plots ranged from 0.24 to 2.78 (**Table 4**). Plot No. 20 had the highest score (2.78), with a diverse species composition, including large-diameter *Castanopsis fissa* and *Endospermum chinense*. Plot No. 3 had the lowest score (0.24), with fewer species and strong interspecific competition, particularly from *V. mangachapoi* and *H. parvifolia*. The plots are ranked by stability index as follows: 20, 15, 9, 18, 6, 10, 19, 5, 14, 17, 13, 8, 7, 12, 1, 4, 16, 11, 2, 3.

**Table 4:** Principal component analysis scores in the community.

Plots No.	*F*_1_	*F*_2_	*F*_3_	*F*_4_	*F*_5_	Stability index
1	-1.24	4.93	3.31	-0.34	0.53	1.68
2	-1.02	4.58	1.80	-0.18	0.26	1.35
3	-0.31	0.34	0.97	0.21	0.17	0.24
4	0.30	3.35	2.85	0.25	0.25	1.67
5	-0.42	5.86	3.07	0.57	0.59	2.28
6	1.07	4.99	3.34	-0.22	-0.05	2.39
7	-0.41	4.61	3.80	0.22	-0.17	1.96
8	0.29	4.60	3.25	-0.10	0.51	2.07
9	-0.30	6.21	3.75	-0.04	0.65	2.48
10	0.07	5.26	4.11	-0.57	1.05	2.35
11	-1.60	3.84	4.20	0.94	0.50	1.60
12	-1.97	5.52	3.13	1.04	1.21	1.82
13	-2.02	6.05	3.61	1.03	1.50	2.08
14	-1.36	5.95	4.24	0.70	0.74	2.27
15	-0.48	5.39	4.27	1.44	1.36	2.54
16	0.40	3.52	2.76	-0.04	-0.18	1.66
17	0.77	5.27	2.53	-0.30	0.13	2.22
18	4.41	3.42	0.18	0.73	-0.46	2.42
19	1.43	4.15	2.65	1.29	-0.16	2.30
20	1.55	5.20	3.49	0.55	0.64	2.78

*F*_1_, *F*_2_, *F*_3_, *F*_4_, and *F*_5_ represent the weighted values of the first, second, third, fourth, and fifth principal components, respectively.

**Figure 5:**
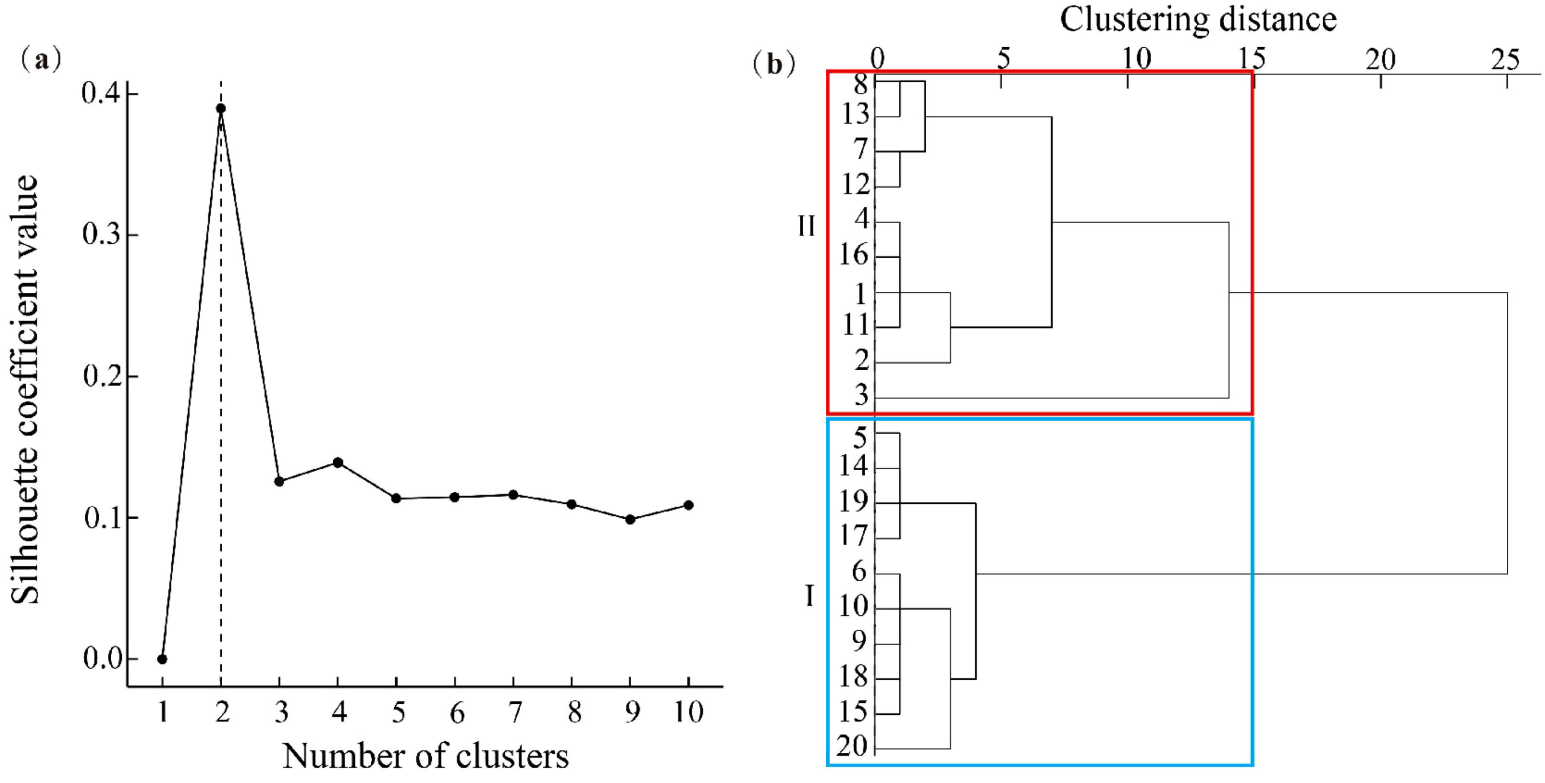
Silhouette optimal number of clusters **(a)**, and stability index of clustering in the community **(b)**. The plots within the blue box belong to the Group I clustering, the plots within the red box belong to the Group II clustering.

Ward clustering of the stability indices, using the silhouette coefficient method, identified two clusters (**Figure 5A**). Group I, with higher stability (stability index 2.22–2.78), includes plots 5, 6, 9, 10, 14, 15, 17, 18, 19, and 20, likely due to lower human disturbance. Group II, with lower stability (stability index 0.24–2.08), includes plots 1, 2, 3, 4, 7, 8, 11, 12, 13, and 16 (**Figure 5B**).

### 3.5 Relationship between community stability and key indicators

**Figure 6:**
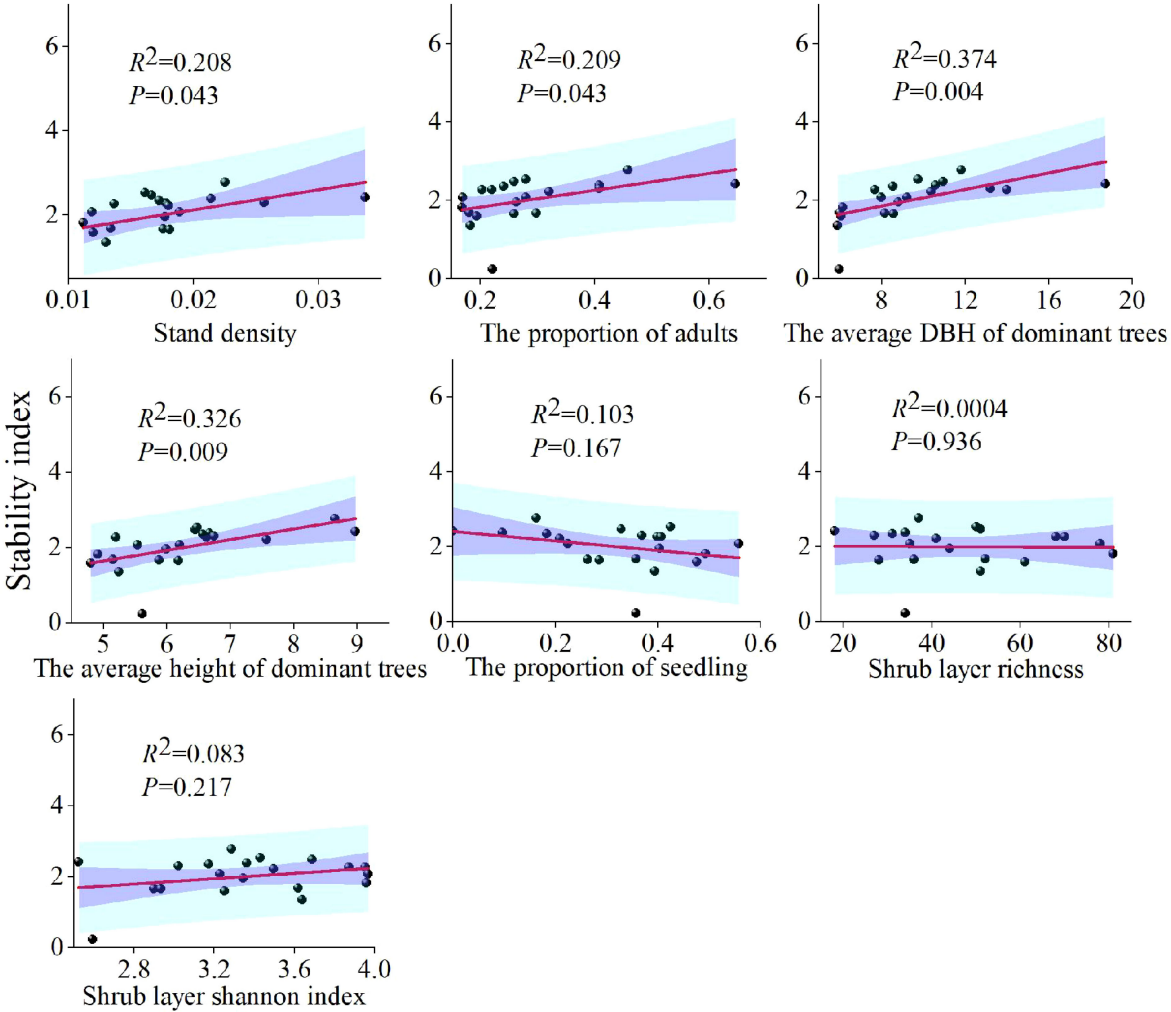
The relationship between stability index and seven key indicators in the community.

Community stability was significantly positively correlated with stand density (*R*² = 0.208, *P* < 0.05), the proportion of arbor adults (*R*² = 0.209, *P* < 0.05), the average DBH of dominant trees (*R*² = 0.374, *P* < 0.05), and the average height of dominant trees (*R*² = 0.326, *P* < 0.05). In contrast, the proportion of arbor seedlings (*R*² = 0.103, *P* > 0.05), shrub layer richness (*R*² = 0.0004, *P* > 0.05), and the shrub layer Shannon index (*R*² = 0.083, *P* > 0.05) showed no significant correlation with stability (**Figure 6**).

### 3.6 Community stability distribution under different terrain conditions

**Figure 7:**
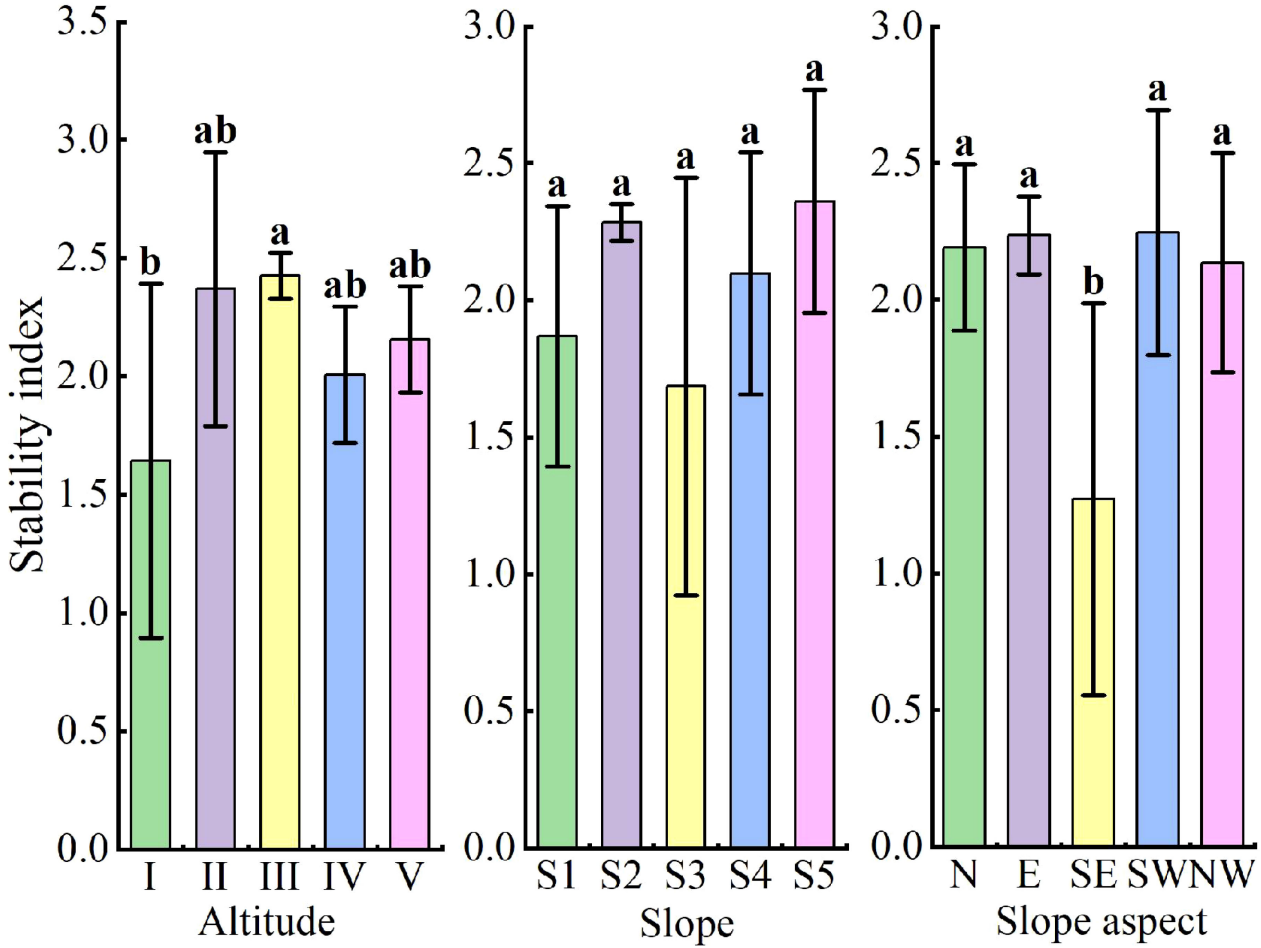
Stability index in the community at different terrain conditions. I: (200–299 m), II: (300–399 m), III: (400–499 m), IV: (500–599 m), V: (600–699 m); S1: (0°–9°), S2: (10°–19°), S3: (20°–29°), S4: (30°–39°), S5: (40°–49°); N: north slope, E: east slope, SE: southeast slope, SW: southwest slope, NW: northwest slope. Different lowercase letters between (a, b, ab) represent significant differences.

Significant differences in stability index were observed across altitudes, with altitude III (400–499 m) having a significantly higher stability index than altitude I (200–299 m) (*P* < 0.05). The stability index on the southeast slope was significantly lower compared to other slope aspects (*P* < 0.05), but slope had no significant effect on community stability (**Figure 7**).

## 4 Discussion

### 4.1 Species composition and structural characteristics

In this study, the community with *H. parvifolia* in the 0.8 ha plot of Diaoluo Mountain, Hainan, exhibited a higher species richness (302 species) compared to similar lowland rainforests, such as the 1.6 ha plot in central Vietnam (170 species) (Van and Cochard, 2017). These differences are primarily due to varying levels of human disturbance and natural disasters. The Diaoluo Mountain area, though previously affected by slash-and-burn farming, has benefited from improved ecosystem stability due to increased ecological awareness and the establishment of national parks. In contrast, Vietnam’s lowland rainforests are influenced by agricultural land and fragmented forests (Yirga et al., 2019). The community is primarily composed of tree species belong to Rubiaceae, Euphorbiaceae, and Lauraceae, contrasting with the surrounding community around the endangered *Cephalotaxus hainanensis*, which is dominated by Moraceae, Lauraceae, and Meliaceae (Wang et al., 2023). These variations highlight the need for region-specific conservation strategies. In the competition for resources, *V. mangachapoi* and *H. parvifolia* are key dominant species, shaping the community structure. The mixed forest of these species in lowland areas of southeastern Hainan has become the primary plant community type.

The shrub layer was found to have the highest species richness. This could be attributed to past logging disturbances, where pioneer species such as *P. asiatica* and *G. oblongifolia* played a crucial role in community recovery. These species’ rapid growth and reproductive capabilities facilitate their dominance during early succession stages (Martínez-Ramos et al., 2021). As the community matures, species diversity increases, enhancing functional diversity and ecosystem stability (Campbell et al., 2011). In contrast, the herbaceous layer remains sparse due to competition for light from the upper layers, poor nutrient conditions in the soil, and allelopathy from rapidly growing shrubs (Edy et al., 2020; Chou et al., 2018).

### 4.2 Stability analysis

In this study, the first principal component, which accounted for the largest contribution to community stability, was heavily influenced by species diversity and stand structure. Factors such as stand density, the average DBH and height of dominant trees, and the proportions of arbor seedlings and adults played critical roles in community stability. These findings align with previous studies on plant community stability (Ullah et al., 2021; Hishe et al., 2021; Wang et al., 2022). An optimal stand density directly impacts resource allocation, while high species diversity enhances resilience to external environmental disturbances. A stable community age structure ensures sustainable regeneration capacity. These factors are crucial in driving material cycling and energy flow processes (Sabatini et al., 2013).

It is impossible to overlook how human involvement affect the stability of communities (Li et al., 2024). The results of this study show that plots in Group I are more stable than Group II. This could be because plots in Group II with a certain level of human influence have more shrubs and intense interspecific competition, whereas plots in Group I with minimal human interference have more large-diameter individuals and well-preserved ecosystems. At the same time, certain plots in Group II at lower altitudes are traversed by tourist roads and rainforest plank roads, which leads to habitat fragmentation. Human activities, such as walking and vehicular traffic, can negatively impact the stability of forest ecosystems. These activities contribute to habitat destruction by reducing soil porosity and raising micro-environmental temperatures, which can hinder seed germination and plant growth (Chisholm and McCune, 2024). Furthermore, they disrupt ecological processes by interfering with pollination and seed dispersal and may facilitate the spread of invasive plant species (Jo et al., 2024). Additionally, field investigations have shown that the cultivation of economic plants such as *A. catechu* and *Artocarpus heterophyllus*, continues within the study area, hindering efforts to restore forest cover. It is crucial for local authorities to enhance law enforcement measures to address this issue.

Although species diversity often correlates with ecosystem stability (Yang et al., 2017), this study found no significant correlation between the species diversity index and community stability. Unlike studies on grassland communities (Ouyang et al., 2021), where the relatively low number of plant species makes changes in species diversity more likely to influence ecosystem stability, tropical rainforests are characterized by high species richness and significant redundancy in key functional traits, such as photosynthetic pathways and water use efficiency (Radford-Smith et al., 2024). The loss of a single species is often compensated by others with similar functions. Furthermore, most tree species in tropical rainforests show weak interspecific relationships and tend to follow an independent distribution pattern (Shang et al., 2025). As a result, changes in species diversity have a minimal effect on the stability of the ecosystem. Additionally, the small size of survey plots may lead to an overestimation of the effects of diversity. This study found a significant positive correlation between stand density, the proportion of adult trees, the average DBH and height of dominant trees with the stability of the community. This suggests that the stability of the community was more closely related to the structural characteristics of dominant tree species, which help regulate regeneration and contribute significantly to stability (Prohaska et al., 2023). It is noteworthy that the study area has experienced logging disturbances, with generalist tree species being widely distributed in lowland rainforests. While biodiversity recovers rapidly, functional diversity recovery lags, and stability may rely more on the structure of the stand structure.

### 4.3 Terrain factors on community stability

The stability index was significantly higher at altitudes of 400–499 m compared to 200–299 m, suggesting that mid-altitude regions provide more favorable water and nutrient conditions for tree growth (Jiang et al., 2023). In contrast, the southeast slope had lower stability due to heightened interspecific competition, intensified by the prevailing monsoon, high light intensity, and rapid soil water evaporation. However, slope did not significantly affect community stability, similar to tropical forests in Central America (Hightower et al., 2014). Adaptations of dominant trees like *H. parvifolia* and *V. mangachapoi*, such as buttress roots, enable them to thrive despite slope variations, enhancing community stability.

### 4.4 Protection strategies

To protect endangered plant communities and restore tropical lowland rainforest vegetation, the following strategies can be implemented:

1. Minimize damage from tourist paths, preserve endangered species in their habitats, and use artificial propagation with careful reintroduction.
2. Prioritize the replanting of dominant tree species and selectively prune generalized plants to promote understory growth.
3. Implement an intelligent monitoring system to detect and address illegal logging activities.
4. Raise ecological awareness, promote farmland-to-forest initiatives, and actively restore forested areas.

### 4.5 Limitations and prospects

While this study offers valuable insights into the impact of community structure and environmental factors on plant community stability, it has limitations in spatial scale and community dynamics exploration. Future research should expand plot sizes, incorporate long-term monitoring, and consider additional indicators such as functional traits, phylogenetic diversity, ecological interactions, and soil factors. Developing a multi-scale, multi-dimensional research framework will provide deeper insights into the stability of endangered plant communities, focusing on their dynamic characteristics.

## 5 Conclusions

This study surveyed the community with *H. parvifolia* to examine its structure and stability characteristics. The key findings are as follows:

1. The community is species-rich, with Rubiaceae being the most diverse family and *Syzygium* the most diverse genus. The community exhibits a clear hierarchical structure, with higher species diversity in the arbor and shrub layers and lower diversity in the herbaceous layer. *V. mangachapoi* and *H. parvifolia* dominate resource competition.
2. Group I plots, with less human interference, show higher stability than Group II plots, which experience more human disturbance. Key factors influencing stability include stand density, the proportion of adult trees, and the average DBH and height of dominant trees.
3. Community stability is significantly higher at altitudes of 400–499 m compared to 200–299 m, while the southeast slope shows significantly lower stability. However, slope overall does not significantly affect stability.

These findings contribute to understanding the community structure and stability of *H. parvifolia*, offering valuable data and theoretical support for the conservation of endangered plants and biodiversity in tropical reserves in China and globally.

## Data Availability

The original contributions presented in the study are included in the article/supplementary material. Further inquiries can be directed to the corresponding authors.
